# Supplementary material for: Computed tomography analysis of guinea pig bone: architecture, bone thickness and dimensions throughout development
Source: PeerJ. 2014 Oct 2;2:e615. doi: 10.7717/peerj.615 (PMC4185290; doi:10.7717/peerj.615)
Supplement: Supplemental Tables — Manual measurements aided by micro-CT. Table S2. Micro-CT scapula measurements. Table S3. Micro-CT humerus measurements. Table S4. Micro-CT femur measurements. Table S5. Micro-CT bone local thickness measurements. [file peerj-02-615-s001.docx]

|  | Scapula measurement (mm) | | | | Humerus measurement (mm) | | | Femur measurement (mm) |
| --- | --- | --- | --- | --- | --- | --- | --- | --- |
| Age | 1 | 2 | 3 | 4 | 1 | 2 | 3 | 1 |
| <1m | 14.78692 ± 0.681408 | 13.88375 ± 0.129751 | 8.95825 ± 0.099666 | 3.414 ± 0.138377 | 18.91225 ± 0.222885 | 4.64225 ± 0.056201 | 4.984625 ± 0.082223 | 19.2245 ± 0.067505 |
| <3m | 18.91725 ± 0.113828 | 16.2575 ± 0.953885 | 10.6355 ± 0.1594 | 4.1015 ± 0.051029 | 22.836 ± 0.232853 | 6.00325 ± 0.178892 | 6.007583 ± 0.141914 | 24.324 ± 0.296283 |
| <6m | 23.73825 ± 0.432808 | 22.87525 ± 0.039202 | 13.908 ± 0.099761 | 5.08575 ± 0.172656 | 25.718 ± 0.243722 | 6.739 ± 0.054169 | 6.2946 67 ± 0.194647 | 31.91108 ± 0.493804 |
| <1yr | 36.24425 ± 1.423744 | 32.42675 ± 0.76535 | 22.04825 ± 1.20107 | 6.87275 ± 0.182466 | 36.327 ± 0.583305 | 8.69225 ± 0.145283 | 7.033667 ± 0.129328 | 45.27675 ± 0.19415 |
| <4yr | 39.11775 ± 0.643163 | 36.22425 ± 0.047149 | 24.697 ± 0.662543 | 7.1505 ± 0.161249 | 39.23525 ± 0.960402 | 9.126 ± 0.197347 | 7.6565 ± 0.044005 | 46.645 ± 0.17619 |

Table S1. Manual measurements aided by micro-CT

Measurements of guinea pig bones aged 0-1 month (<1m), 1-3 months (<3m), 3-6 months (<6m), 6 months-1 year (<1yr) and 1-4 years (<4yr). Mean ± standard error of the mean.

Table S2. Micro-CT scapula measurements

| Age | Width (mm) | Depth (mm) | Length (mm) | Volume (mm^3^) | Surface Area (mm^2^) |
| --- | --- | --- | --- | --- | --- |
| <1m | 8.8605 ± 2.009764062 | 6.723333333 ± 0.732380385 | 15.628 ± 1.174507982 | 48.26333 ± 15.42707 | 494.5083333 ± 39.57178544 |
| <3m | 13.376 ± 1.266 | 9.735 ± 0.495 | 19.124 ± 0.221 | 84.035 ± 5.035 | 853.225 ± 12.995 |
| <6m | 12.50883333 ± 0.560374304 | 9.422 ± 0.425770282 | 25.0296667 ± 1.41154647 | 115.1867 ± 10.49336 | 1058.628333 ± 167.3813227 |
| <1yr | 18.85216667 ± 2.454374162 | 17.4381667 ± 3.62959176 | 37.757 ± 1.039526375 | 274.3917 ± 41.19161 | 2550.641667 ± 126.7607018 |
| <4yr | 25.13275 ± 0.16375 | 15.16325 ± 1.90325 | 41.0235 ± 0.9635 | 367.02 ± 17.28 | 3304.605 ± 70.485 |

Scapula measurements aged 0-1 month (<1m), 1-3 months (<3m), 3-6 months (<6m), 6 months-1 year (<1yr) and 1-4 years (<4yr). Mean ± standard error of the mean error bars.

Table S3 Micro-CT humerus measurements.

| Age | Width (mm) | Depth (mm) | Length (mm) | Volume (mm^3^) | Surface Area (mm^2^) |
| --- | --- | --- | --- | --- | --- |
| <1m | 6.2592 ± 1.054599955 | 6.509 ± 1.062495729 | 17.6822 ± 1.035842696 | 66.527 ± 13.09796 | 509.77 ± 91.01475651 |
| <3m | 7.73925 ± 0.64875 | 7.56975 ± 0.56375 | 25.321 ± 1.612 | 117.71 ± 24.13 | 1110.495 ± 29.69 |
| <6m | 9.535166667 ± 1.394003657 | 7.297 ± 0.314366559 | 27.248 ± 1.601946498 | 150.8917 ± 26.87475 | 1153.57 ± 64.82247784 |
| <1yr | 9.308833333 ± 1.622119096 | 10.412 ± 1.0185063 | 36.25783333 ± 2.028957908 | 290.5417 ± 33.34097 | 2246.638333 ± 282.6312533 |
| <4yr | 12.626 ± 1.688349218 | 10.9575 ± 0.90029472 | 39.68433333 ± 1.064083421 | 322.425 ± 16.91729 | 2751.45 ± 438.9385857 |

Humerus measurements aged 0-1 month (<1m), 1-3 months (<3m), 3-6 months (<6m), 6 months-1 year (<1yr) and 1-4 years (<4yr). Mean ± standard error of the mean.

Table S4 Micro-CT femur measurements.

| Age | Width (mm) | Depth (mm) | Length (mm) | Volume (mm^3^) | Surface Area (mm^2^) |
| --- | --- | --- | --- | --- | --- |
| <1m | 5.95526 ± 0.336318402 | 7.0086 ± 0.695762053 | 19.0831 ± 1.033349111 | 89.408 ± 18.17169 | 677.484 ± 121.1086005 |
| <3m | 8.64825 ± 1.14525 | 8.10275 ± 0.87875 | 26.84175 ± 0.96375 | 152.7175 ± 14.0075 | 1600.0925 ± 13.9475 |
| <6m | 9.523 ± 0.113892859 | 9.886833333 ± 0.460022675 | 30.62583333 ± 2.766904232 | 220.2117 ± 54.64612 | 1637.306667 ± 245.8208254 |
| <1yr | 10.0545 ± 1.618 | 10.69075 ± 0.81225 | 41.99925 ± 4.76375 | 401.555 ± 174.57 | 2579.9125 ± 784.3775 |
| <4yr | 13.47833333 ± 1.362279014 | 12.46033333 ± 2.623746944 | 45.76716667 ± 1.48593619 | 627.2583 ± 54.01982 | 4096.178333 ± 457.158429 |

Femur measurements aged 0-1 month (<1m), 1-3 months (<3m), 3-6 months (<6m), 6 months-1 year (<1yr) and 1-4 years (<4yr). Mean ± standard error of the mean.

Table S5 Micro-CT bone local thickness measurements.

|  | Scapula Average | | Humerus Average | | Femur Average | |
| --- | --- | --- | --- | --- | --- | --- |
| Age | Mean thickness (mm) | Max thickness (mm) | Mean thickness (mm) | Max thickness (mm) | Mean thickness (mm) | Max thickness (mm) |
| <1m | 0.18 ± 0.02 | 0.55 ± 0.02 | 0.25 ± 0.02 | 0.62 ± 0.05 | 0.22 ± 0.01 | 0.61 ± 0.05 |
| <3m | 0.29 ± 0.01 | 0.71 ± 0.06 | 0.31 ± 0.09 | 0.73 ± 0.19 | 0.29 ± 0.04 | 0.78 ± 0.12 |
| <6m | 0.25 ± 0.01 | 0.64 ± 0.07 | 0.36 ± 0.08 | 0.83 ± 0.21 | 0.39 ± 0.05 | 0.97 ± 0.11 |
| <1yr | 0.35 ± 0.03 | 0.87 ± 0.09 | 0.41 ± 0.05 | 0.89 ± 0.08 | 0.52 ± 0.06 | 1.22 ± 0.07 |
| <4yr | 0.29 ± 0.03 | 0.85 ± 0.05 | 0.45 ± 0.05 | 0.96 ± 0.07 | 0.54 ± 0.03 | 1.39 ± 0.04 |

Measurements of guinea pig scapula, humerus and femur thickness aged 0-1 month (<1m), 1-3 months (<3m), 3-6 months (<6m), 6 months-1 year (<1yr) and 1-4 years (<4yr). Mean ± standard error of the mean.
