# Supplementary material for: Computed tomography analysis of guinea pig bone: architecture, bone thickness and dimensions throughout development
Source: PeerJ. 2014 Oct 2;2:e615. doi: 10.7717/peerj.615 (PMC4185290; doi:10.7717/peerj.615)
Supplement: Supplemental Information 2 [file peerj-02-615-s002.rtf]

The University of
Nottingham


06/06/14
Dear Sir or Madam
The project entitled: 'Common conditions affecting the guinea pig: breed predispositions and disease morphology' was reviewed in May 2012 by the School of Veterinary Medicine and Science (SVMS) Ethical Review Committee, chaired by Professor David Haig. The study passed ethical review and was allowed to proceed. As the project involved material taken from animals post-mortem, the study fell neither under the Veterinary Surgeons Act (UK) nor the Animals, Scientific Procedures Act (ASPA, UK). The proposer of the study and principal investigator is Dr Catrin Rutland of the SVMS. The SVMS ethical review committee consists of research scientists and clinicians as well as lay members and ethically reviews all research projects at the SVMS, University of Nottingham. 

I hope that this is sufficient for your purposes.

Yours faithfully,


Professor David Haig,  B.Sc., M.Sc., Ph.D.
Chair, Animal Infection and Immunity; Chair, SVMS Ethical review Committee.
